# Cost analysis of pediatric intensive care: a low-middle income country perspective

**DOI:** 10.1186/s12913-021-06166-0

**Published:** 2021-02-23

**Authors:** Amrit Kaur, Muralidharan Jayashree, Shankar Prinja, Ranjana Singh, Arun K. Baranwal

**Affiliations:** 1 Division of Pediatric Critical Care, Department of Pediatrics, Advanced Pediatrics Centre, Post Graduate Institute of Medical Education & Research, Chandigarh, India; 2 Department of Community Medicine and School of Public Health, Post Graduate Institute of Medical Education & Research, Chandigarh, India; 3 Department of Hospital Administration, Advanced Pediatrics Centre, Post Graduate Institute of Medical Education & Research, Chandigarh, India

**Keywords:** Cost analysis, Expenditure, Pediatric intensive care, Tertiary care

## Abstract

**Background:**

Globally, Pediatric Intensive Care Unit (PICU) admissions are amongst the most expensive. In low middle-income countries, out of pocket expenditure (OOP) constitutes a major portion of the total expenditure. This makes it important to gain insights into the cost of pediatric intensive care. We undertook this study to calculate the health system cost and out of pocket expenditure incurred per patient during PICU stay.

**Methods:**

Prospective study conducted in a state of the art tertiary level PICU of a teaching and referral hospital. Bottom-up micro costing methods were used to assess the health system cost. Annual data regarding hospital resources used for PICU care was collected from January to December 2018. Data regarding OOP was collected from 299 patients admitted from July 2017 to December 2018. The latter period was divided into four intervals, each of four and a half months duration and data was collected for 1 month in each interval. Per patient and per bed day costs for treatment were estimated both from health system and patient’s perspective.

**Results:**

The median (inter-quartile range, IQR) length of PICU stay was 5(3–8) days. Mean ± SD Pediatric Risk of Mortality Score **(**PRISM III) score of the study cohort was 22.23 ± 7.3. Of the total patients, 55.9% (167) were ventilated. Mean cost per patient treated was US$ 2078(₹ 144,566). Of this, health system cost and OOP expenditure per patient were US$ 1731 (₹ 120,425) and 352 (₹ 24,535) respectively. OOP expenditure of a ventilated child was twice that of a non- ventilated child.

**Conclusions:**

The fixed cost of PICU care was 3.8 times more than variable costs. Major portion of cost was borne by the hospital. Severe illness, longer ICU stay and ventilation were associated with increased costs. This study can be used to set the reimbursement package rates under Ayushman Bharat – *Pradhan Mantri Jan Arogya Yojana* (AB-PMJAY). Tertiary level intensive care in a public sector teaching hospital in India is far less expensive than developed countries.

## Background

Globally, Pediatric Intensive Care Unit (PICU) admissions are amongst the most expensive of all hospital admissions. They significantly impact the financial dynamics of patient’s families. Although intensive care beds comprise only about 10% of hospital beds, they account for 20–40% of all hospital costs especially in the high income countries [1]. However, health insurance coverage and availability of drugs and consumables from hospital supplies ease financial burden for patient’s families. The scenario in low middle-income countries (LMIC) like India is different; cost of health care services is borne by both government as well as patients. According to National Health Accounts guidelines of our country, out of pocket expenditure (OOP) constitutes 68 to 70% of the total expenditure [2]. The OOP for families whose children are admitted to intensive care units is very high. The Indian government launched the Ayushman Bharat – *Pradhan Mantri Jan Arogya Yojana* (AB-PMJAY), a health insurance scheme in September 2018 with the aim to cover 50 crore people [3]. However, the health benefit package (HBP) rates in this scheme are low due to lack of reference on costing data in India. The existing packages were determined through a consultative process with experts and a review of existing health insurance schemes. A problem with such charge-based reimbursement rates is that they do not capture the actual cost and resource use. Health Technology Assessment India (HTAIn) commissioned a national costing study entitled ‘Cost of Health Services in India’ (CHSI) for information regarding reimbursement rates of HBPs under AB - PMJAY and for use in economic evaluations [4]. PM – JAY focuses on costing data from Level III care as Level II estimates are already available.

Although there have been few studies from India on cost analysis of neonatal intensive care, cost analysis of PICU care and the financial impact on the families of children receiving such care in India is limited.

Our multispecialty public sector pediatric hospital caters to a large volume of referrals from 5 to 6 north western states of our country viz. Haryana, Punjab, Himachal Pradesh, Uttar Pradesh, Jammu &Kashmir and Rajasthan and provides comprehensive care to these children. The PICU in our hospital offers state of the art Level III intensive care services to critically ill children referred from the regions.

Cost analysis from such a set up looking at both health system as well as patient’s perspective would provide insight into the financial burden and its impact on hospital and communities.

Also, the data generated through this study will help in budget planning, allocation, effective utilisation of resources and can serve as a benchmark for other similar ICUs. More importantly, in the context of economic evaluations, costing studies from tertiary care centres like ours can help in revising rates of many health benefit packages.

## Methods

### Study setting

We undertook this prospective observational cross-sectional study in our 15 bedded Level III PICU which has an annual admission average of 900 to 1000 patients. It has state of the art respiratory, hemodynamic and multimodal neuromonitoring facilities. Diagnostics such as echocardiography, X-ray, ultra-sonography, and arterial blood gas monitoring are available round the clock. While provisions for intracranial pressure monitoring, haemodialysis and continuous renal replacement therapy exist, ECMO is not available. A total of 912 patients were admitted in the PICU during 2018.

### Study design

In low middle-income countries, cost of health care services is borne by both government as well as patients. According to National Health Accounts guidelines of our country, OOP constitutes 68 to 70% of the total expenditure [2]. Therefore, we undertook this study from a societal perspective and estimated both health system and out of pocket expenditure to assess the overall cost of provision of intensive care services.

#### Health system cost

We used a combination of both top down and bottom up approach for micro costing [5–9]. The cost of human resources, infrastructure, equipment, physical space and overheads were estimated using the top down approach. Costing of drugs and consumables and diagnostics was calculated using the bottom up method.

#### Resource identification

Annual data regarding hospital resources utilized for the PICU care was collected from January 2018 to December 2018. We chose 1 year period to iron out any difference in volume and type of patients who access these services. All resources were classified as recurrent and capital resources. Recurrent costs are the costs of maintaining and operating a unit once the initial investment has been completed. Human resources, diagnostics, consumables and overhead costs (i.e. water consumption, electricity and dietetics etc.) comprised recurrent costs. Capital costs occur one time and are unlikely to recur again. Costs of building or space, medical and non - medical equipment and furniture were part of capital costs.

#### Resource measurement

The methodology for data collection included reviewing records, staff interviews and physical inspection of facility. The collected data included number of human resources (i.e. medical and paramedical staff, administrative and support staff), space in the building, number and types of equipment, other non-consumable items, diagnostic tests (laboratory and radiological), medicines, consumables and other overhead costs. Data was collected on the quantity of different resources being exclusively used in PICU.

Staff members were interviewed to obtain information on the time spent by them in PICU and further annual salary was apportioned to number of working days in PICU. Pretested questionnaires used in previous Indian studies were used [10–13]. Stock registers of PICU served as major source for data regarding quantities of consumables and non-consumables and annual cost was calculated by annual amount and price data. The actual cost of diagnostic services was 3.04 times higher than what was charged to patients and hence calculated by multiplying each investigation charge by a factor (3.04) thereby converting the user fee into total cost of diagnostics [14]. Data for the overhead costs was collected by record review of electricity bills and water bills.

#### Resource valuation

Hospital’s procurement branch had provided the data regarding capital costs, including the year of purchase and price of medical and non-medical equipment [10, 14]. Technical officer in – charge of PICU was interviewed to elicit information regarding average life of the equipment. Apart from this, literature was reviewed to cross validate for the life of capital items.

#### Out of pocket expenditure

Data regarding expenditure borne by the patient families for bed charges, medicines, and investigations for their ward’s treatment in PICU and transportation and meals of attendants was collected by a daily interview till the discharge or transfer or final outcome of the patient. In order to have data representative of entire duration, the study period of one and a half years from July 2017 to December 2018, was divided into four intervals, each of 4 and a half months duration. Data was collected for 1 month in each interval wherein children > 1 month to 12 years admitted in PICU during that interval were enrolled consecutively.

Written and informed consent was obtained from the parents before eliciting information related to OOP. We also collected data on socio-demographic characteristics of the families of the patient admitted, which included education and occupation of the head of the family and per capita income. Information regarding methods to cope with treatment expenses and salary losses were elicited using questionnaire.

To correlate cost with severity of illness, Pediatric Risk of Mortality Score **(**PRISM III Score) validated from Physiologic Stability Index (PSI) was calculated for every patient within 24 h of admission to PICU [15]. Patients were divided into 5 groups based on their PRISM III Score such as 0–10, 11–20, 21–30, 31–40, 41 and above. Subsequently average total out of pocket expenditure per patient within each group was calculated.

### Data analysis

#### Health system costs

Data analysis was done using Statistical Package for Social Sciences (SPSS) version 25 and MS Excel. The cost of the PICU space / building was estimated by applying rental price for the area. Annualized costs of various medical and non-medical equipment in the PICU were estimated based on their average useful life and application of an average discount rate. A discount rate of 3% was used to compute the annualization factor [16]. Methodological guidelines for conduct of economic evaluation published by the Department of Health Research in India recommend a 3% discount rate for valuing future costs and outcome [17]. In the systemic review of economic evaluation in India, 3% discount rate was accounted as the most prevalent practice adopted by them [18]. Further, most recent costing studies and economic evaluation in India have recommended and used 3% discount rate for computing annual cost [19–21]. All the cost estimates were finally converted to 2018 prices to adjust for inflation, applying a discounting factor of 3% per year.

#### Apportioning statistics

Appropriate apportioning statistics were used to allot shared or joint resources to the PICU. Firstly, for the human resources, we interviewed people involved in PICU services, which mainly included consultants, residents, nursing officers and technical staff to obtain data on patterns of work flow and time devoted to PICU. Proportion of time spent in PICU care was used to apportion the shared human resource costs.

Secondly, costs of shared building/space (i.e. waiting hall, discussion room, residents’ room, nursing station, laboratory etc.), overheads and equipment were apportioned on the basis of bed-days of PICU patients in a year. Cost of medicines and consumables was estimated for PICU alone and hence this was not a shared cost so there was no requirement of apportioning.

#### Unit costs

The health system costs were summated and calculated as Annual Health System Cost and then divided by the total number of patients admitted in the study year (2018) to calculate the average health system cost per patient. Sources of data are described in Table 1.

**Table 1:** Sources of data and apportioning statistics for Pediatric Intensive care unit type of data

	Source of data	Form of data	Method used to analyse/estimate annual cost	Allocation criteria
**Non Recurrent/Capital items**				
Building/Space	Record review (maps), facility, observation	Maps	Estimated the floor size of constructed area multiplied with local commercial rental price	Record taken from civil engineering department of tertiary hospital and shared areas apportioned on the basis of number of patients
Equipment	Record review (stock register), facility observation	Stock registers	Annualization factor multiplied with purchase price plus annual maintenance cost	Equipment costs were designated by the centre
**Recurrent items**				
Human Resource	Record review, Interview, Facility, observation	Pay slips, on call books	Salary multiplied with the proportion of time spent in a year on PICU care services	Most of the human resources were providing full time of their duty to PICU care except some nursing officers and on call doctors for whom apportioning was done on the basis of time allocated to PICU care
Medicines and consumables	Record review	Stock register	Annual amount of drugs/consumables and price data	Amount of medicines and consumables was taken separately for PICU
Other consumables (stationary)	Record review	Stock register	Annual amount of consumables and price data	Amount of other consumables was taken separately for PICU
Diagnostics	Record review of cases	Investigations charge slips	User fee multiplied with a factor (3.04)	Diagnostic costs were designated by the centre
**Overheads**				
Electricity	Record review	Monthly electricity bills	Annual consumption of electricity in PICU	Apportioning was done on the basis of floor area of PICU
Water	Record review	Monthly water bills	Annual consumption of water in PICU	Apportioning was done on the basis of floor area of PICU
Annual maintenance (overall)	Record review	Stock register	Annual amount spent on consumption	Apportioning was done on the basis of floor area of PICU
Sanitation	Record review	Stock register	Annual amount spent on consumption	Amount of sanitary items was taken separately for PICU
Dietetics	Record review	Stock register	Annual volume of diets	Amount of diets consumed was taken separately for PICU

Further, per patient health system cost was calculated by dividing total annual cost by total bed days of length of stay per patient.

#### Out of pocket expenditure

We estimated the mean and standard error of OOP expenditure from the data collected from the patients. Descriptive statistics [frequencies and proportions, mean ± standard deviation (SD) and Median (IQR)] were used for baseline data analysis. Chi Square test, Fisher exact test and Mann – Whitney test were used to calculate relation between relevant parameters.

### Sensitivity analysis

Univariate sensitivity analysis was undertaken to evaluate the influence of parameter uncertainties on total costs. Salaries for human resources, prices of drugs, equipment, and rental costs were varied by 25, 50, 25 and 25% respectively on either side of the base value. In order to assess the effect of discount rate on the total annual cost of pediatric intensive care, the discount rate was also varied from 0 to 5%.

## Results

### Sample characteristics

A total of 299 children were enrolled for the estimation of OOP. The median (IQR) age of the study subjects was 4 years (0.8 to 12 years). Of all patients, boys represented 63.5% (190). The median (IQR) length of PICU stay was of 5 days (3–8 days). Nearly half of the parents had completed middle and high school education. Only 21(7%) and 7(2.3%) were graduates and post graduates respectively. Socio economic status classification according to Kuppuswami Scale 2018, showed that 288 (96.5%) of admitted children belonged to the lower middle and upper lower socio-economic strata. Out of them 49.8% were labourers and daily wage workers, 44.8% were self-employed and only 5% were salaried personnel. Around 85% patients were discharged or transferred to wards from PICU, while forty-six patients had died (Table 2). The mean ± SD PRISM III score was 22.2 ± 7.3 with maximum score being 46. Common indications for PICU admission were respiratory (40.8%), followed by hemodynamic needs (19.7%) and combined in the rest. Among the diagnostic categories, tropical fevers (dengue, scrub typhus, malaria, enteric fever) were the commonest (*n* = 51; 17%), followed by community acquired pneumonia (*n* = 50; 16.7%), acute meningoencephalitis (*n* = 42; 14%), disseminated staphylococcal sepsis (*n* = 32; 10.7%), acute bronchiolitis (*n* = 20; 6.6%), poisoning and envenomation (*n* = 19; 6.3%), acute meningitis(*n* = 13; 4.3%), acute gastroenteritis(*n* = 10; 3.3%) and others (*n* = 69; 20.7%) (Table 4).

**Table 2:** Characteristics and out of pocket expenditure of patients admitted to Level III PICU

Characteristics	US$ (₹)			
	*N*	%	Mean	SE
Gender				
Male	190	63.5	368 (25,659)	28 (1965)
Female	109	36.5	324 (22,576)	17 (1191)
Total	299	100	352 (24,535)	19 (1323)
Age group				
< 5 years	172	57.5	334 (23,260)	15 (1087)
> 5 years	127	42.5	377 (26,262)	39 (2745)
Total	299	100	353 (24,535)	19 (1323)
Education				
Illiterate	16	5.4	444 (30,914)	44 (3128)
Primary school	103	34.4	335 (23,360)	19 (1334)
Middle school	82	27.4	314 (21,883)	22 (1598)
High school	68	22.7	423 (29,430)	71 (4957)
Diploma	2	0.7	380 (26,450)	0
Graduate	21	7.0	293 (20,453)	33 (2298)
Professor of Honors	7	2.3	322 (22,466)	49 (3457)
Total	299	100	352 (24,535)	19 (1323)
Kuppuswami scale				
Upper Middle	11	3.7	288 (20,096)	59 (4138)
Lower Middle	122	40.8	370 (25,786)	41 (2883)
Upper Lower	166	55.5	343 (23,910)	15 (1063)
Total	299	100	352 (24,535)	19 (1323)
Occupation				
Labourer	149	49.8	329 (22,926)	15 (1068)
Self-employed	134	44.8	342 (23,828)	18 (1292)
Salaried	15	5.0	644 (44,819)	303 (21,114)
Unemployed	1	0.3	788 (54,830)	0
Total	299	100	352 (24,535)	19 (1323)
PRISM SCORE				
11–20	148	49.5	235 (16,376)	12 (8,30)
21–30	100	33.4	413 (28,765)	18 (1307)
31–40	48	16.1	597 (41,552)	94 (6599)
> 41	3	1	198 (13,836)	34 (2374)
Total	299	100	352 (24,535)	19 (1323)
Ventilated	167	55.9	466 (32,482)	30 (2118)
Non ventilated	132	44.1	208 (14,482)	9 (67,2)
Total	299	100	352 (24,535)	19 (1323)
Duration of ventilation				
< 2 days	154	51.5	210 (14,653)	8 (5,86)
> 2 days	145	48.5	503 (35,032)	34 (2366)
Total	299	100	352 (24,535)	19 (13,23)
Outcome				
Alive	253	84.6	333 (23,208)	21 (1511)
Dead	46	15.4	457 (31,838)	27 (1912)
Total	299	100	352 (24,535)	19 (1323)

Conversion rate: US$1 = ₹ 69.57

*SE* Standard error of the mean, *US$* United states dollar

### Health system costs

The total health system cost incurred to treat 912 children in PICU during the year 2018, was estimated to be US$ 1,578,900 (₹109,844,073). Share of human resources was the highest (58.6%), followed by Capital space (16%) and Equipments (15.6%). The total mean cost per patient treated and per bed-day in the PICU was found to be US$ 2078 (₹144,566) and 415 (₹ 28,871) respectively. Of this, the mean health system cost per patient and per bed day was US$ 1731 (₹120,425) and 346 (₹24,071) respectively. Table 3 depicts Annual and unit health system costs and total costs for patients admitted in PICU.

**Table 3:** Annual health system costs and unit costs of treatment of patients in Level III PICU

Health system costs	US$ (₹)		Percentages
Annual costs			
Human Resources	924,482	(64316004)	58.6%
Physical Space	253,182	(17613871)	16%
Equipment	245,519	(17080756)	15.6%
Diagnostics	101,710	(7075964)	6.4%
Drugs and consumables	34,826	(2422844)	2.2%
Overheads	16,965	(1180255)	1.1%
Non- consumable items	2216	(154167)	0.1%
Total annual health system cost	1,578,900	(109844073)	
Unit costs			
Average total cost per patient	2078	(144566)	
Average total cost per bed - day	415	(28871)	
Average health system cost per patient	1731	(120425)	
Average health system cost per bed day	346	(24071)	

Conversion rate: US$ 1 = ₹ 69.57

*US$* United states dollar

### Out of pocket expenditure

**Fig. 1:**
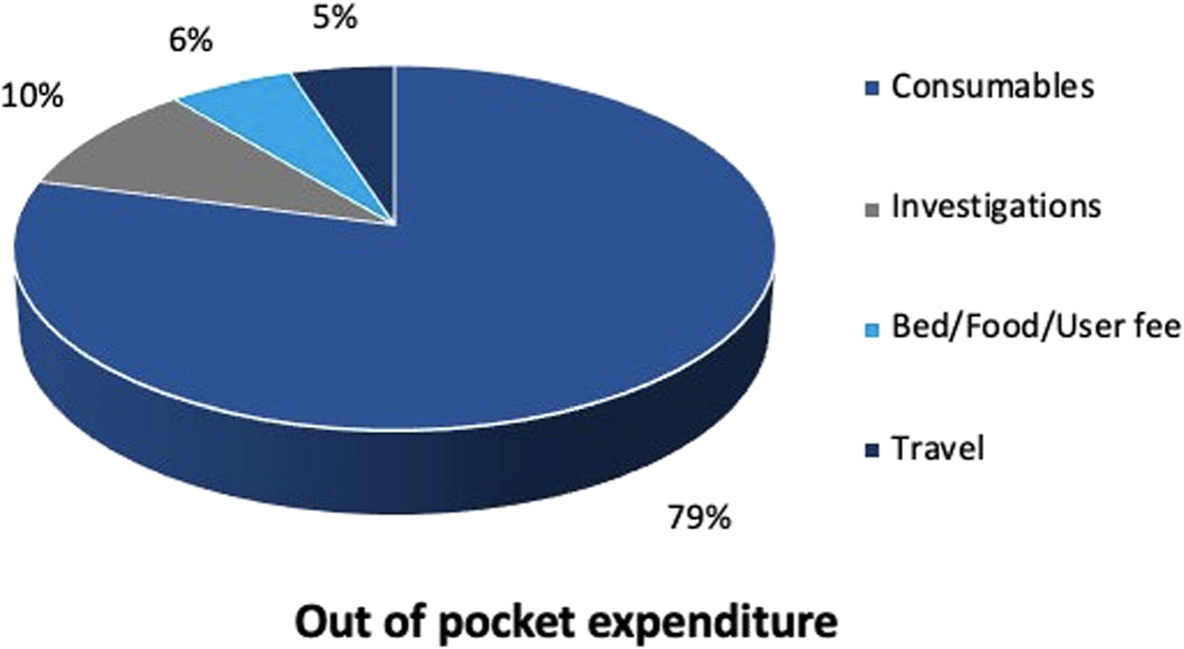
Determinants (%) of out of pocket expenditure of patients admitted in Level III PICU

The mean OOP expenditures for treatment in PICU was US$ 352 (95% CI 315–390) as shown in Table 2. Medicines and consumables accounted for a major share of OOP expenditure i.e. 79% (Fig. 1). Mean OOP expenditures per patient and per patient bed day was US$352 (₹ 24,535) and US$70 (₹4897). Mean OOP expenditures for ventilated patient was significantly higher than a non-ventilated [US$ 466 (₹32,482) vs US$ 208 (₹14,482); *p* < 0.001]. Similarly, the OOP expenditure in patients with PICU stay of ≤2 days was US$ 210 (₹14,653), which was almost one third of the OOP expenditure among patients with PICU stay of > 2 days i.e. US$ 503 (₹ 35,032) (Table 2).

We categorised our study patients into 5 groups based on their PRISM III such as 0–10, 11–20, 21–30, 31–40, 41 and above. Correlating the severity of illness with OOP, we found that average OOP per patient with PRISM III between ‘11 – 20’ was US$ 235 (₹16,376) which was significantly less as compared to patients with PRISM III 21 and above US$ 413 (₹28,765) (Table 2). Additional analysis was undertaken to estimate the mean OOP expenditure for a particular diagnostic category. Out of pocket expenditure was maximum for acute meningoencephalitis ranging from US$ 244 to 707 (₹16,985–49,142). Mean (SE) and Median values are shown in Table 4.

**Table 4:** Diagnostic categories and out of pocket expenditure of patients admitted to level III PICU

Diagnosis	*N*	%	US$		
			Mean	(SE)	Median (IQR)
Tropical Infections	51	17%	327	(204)	240 (175–473)
Acute meningoencephalitis	42	14%	602	(387)	451 (244–707)
Community acquired pneumonia	50	16.7%	362	(199)	291 (216–559)
Disseminated staphylococcal sepsis	32	10.7%	334	(122)	306 (235–397)
Acute bronchiolitis	20	6.6%	176	(73)	154 (131–181)
Poisoning and Envenomation	19	6.3%	288	(130)	237 (216–360)
Acute pyogenic meningitis	13	4.3%	309	(138)	237 (207–454)
Acute gastroenteritis	10	3.3%	502	(289)	462 (264–596)
Others	69	20.7%	425	(252)	364 (234–565)

Conversion rate: US$ 1 = ₹ 69.57

*US$* United states dollar

### Sensitivity analysis

**Fig. 2:**
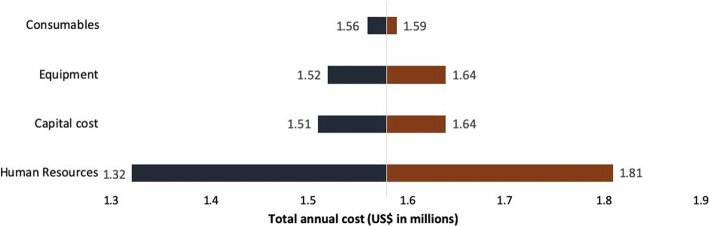
Tornado diagram showing sensitivity analysis

Sensitivity analysis revealed that, total annual costs were most sensitive to variation in human resource costs followed by capital, equipment and medicines and consumables as shown in Tornado diagram (Fig. 2). We also found that variation in discount rate, was associated with an increase of total cost by 20% (undiscounted) to a decrease by16% (discount rate of 5%).

## Discussion

Globally PICU admissions are most expensive and have significant impact on financial dynamics of patient families. India, being a lower middle-income country, financial burden on most of the middle class families is beyond their reach and far more than their family income. Lack of health care insurance and government health care support in most places increases the financial burden further. Our findings revealed that 80% of the total cost incurred to a patient when cared for in PICU was borne by the hospital and only 20% by the patient. This finding is opposite to the general patterns of health care financing wherein nearly 70% of total health care costs are borne by out-of-pocket by patients [22]. Of the health system cost, the major expenditure was towards staff salaries (58.6%) followed by physical space and infrastructure (16%), equipment (15.6%), diagnostics (6.4%), and the rest by drugs and consumables (hospital supply), non-medical equipment charges and overheads. Staff salaries contribute to the major chunk of expenditure because of the higher number of doctors and nurses deployed in PICU. Study by Moerer et al. had also found cost of staffing to be the highest expenditure in intensive care [23]. A study in a neonatal intensive care unit by Narang et al. had also shown similar results; personnel salary constituted 55% of the running costs [10]. A French study by Garcia et al., also had shown much higher proportion of salary cost, 82% of ICU budget was consumed by staff cost [24].

The out of pocket expenditure per patient in our study constituted approximately 20% of the total PICU cost per patient. Although a significant proportion of supplies are provided by the hospital, being a public sector hospital and comparatively cheaper, it is overburdened with large volume of patients. Most patients belonged to the lower middle and upper lower socio-economic strata and more than half were daily wage workers who earned about US$ 100–200 per month. Hence they depend on public sector hospitals for healthcare. Although the OOP, was low compared to international standards, it still translates into significant cost burden for our economically disadvantaged patients.

The OOP in a ventilated patient was double that of a non-ventilated child. Mechanical ventilation requires increased diagnostic and therapeutic procedures, invasive monitoring and drugs and other consumables, thus escalating the cost per patient. Shweta et al. had shown that at all levels of care, the most expensive were those requiring mechanical ventilation [25]. Similar findings were reported in the study by Moerer et al. [23]

We found that the costs of intensive care in PRISM III group of ‘11 – 20’ was significantly less as compared to PRISM III group above 20. However, the cost incurred was similar between PRISM III group ‘21 – 30’, ‘31–40’ and ‘41 and more’. This is explained by the fact that children with PRISM III score above 20 are all severely sick and hence their intensive care needs and cost involved will be similar. Garcia et al. in their study comparing Physiological Stability Index (PSI) with costing had found that increase in PSI on day 1 was associated with increased cost of investigations and treatment [24].

Length of ventilation and PICU stay correlated with the OOP; average OOP in a patient with PICU stay of < 2 days was almost one third the average cost of patients with PICU stay of > 2 days. Similar findings were reported by Chalom et al. where the length of stay had the strongest correlation with the average cost per patient [26].

It has been reported that low reimbursement rates under insurance programmes results in a practice of “balance billing” i.e. the hospitals charge the remaining amount to the patients [27]. This leads to patients continuing to pay out of pocket expenditures for a care which is entitled to be free. As a result empirical cost analysis study like ours will guide policy for price setting [28].

Estimation of health system cost for each diagnostic category and procedures apart from ventilation was not possible due to the methodology of data collection. However, the unit health system cost along with the volume of service utilisation based on standard treatment guidelines and estimates of OOP expenditure for each diagnostic category may help to derive provider payment rates of different packages under (AB-PMJAY) scheme. Furthermore, the indirect cost incurred as a result of lost productivity due to care giver’s time was not measured. None of the government programmes reimburse the indirect costs incurred for treatment.

The Department of Health Research has commissioned a nationally representative study to estimate the cost of health care services for setting the reimbursement rates under AB-PMJAY [3]. The cost estimates from this study published recently showed wide discrepancy between the actual cost of providing services and what is actually paid under the health insurance programme [29]. However, these estimates do not include the cost of providing pediatric services.

There are few published estimates for cost of pediatric intensive care. However these studies are either too dated or have been undertaken in secondary care settings (Level II ICU) [10, 30].

Our study was done in a tertiary level PICU which has one of the best intensive care facilities available at an affordable cost in a public sector hospital. In developing countries, cost of ICU care in public is much less than corporate sector hospitals. A study from another tertiary care teaching hospital in our country had shown similar cost analysis; total cost per patient and per day cost amounted to US$ 1705 and US$ 229 respectively [31]. However, unlike our hospital, the total cost is completely borne by the patient families.

Compared to the cost of intensive care in developed nations, ICU costs are low in the developing countries. A study done by Children’s Hospital of Philadelphia USA, had shown total cost for a patient admitted to PICU amounted to $12,342 while per day per patient cost amounted to $2264 [26]. Similarly, a study from a teaching university hospital of Thessaly, Greece showed mean actual cost per ICU patient to be ∈16,516, actual reimbursement from social funds was only ∈1671 [32]. A study by Moran et al. in Queen Elizabeth Hospital, Australia, on cost calculation in adult intensive care unit showed that the median cost per patient was AUS$2534 (range AUS$106 to AUS$95602) [33]. This low cost of ICU care in India is partly attributed to low cost of drugs, recycling of consumables and lower staff salaries.

To the best of our knowledge, this is the first study of its kind, to address the detailed cost analysis of a tertiary level PICU care in a public sector hospital. The strength of our study lies in our robust cost calculation that has taken into account all the fixed and variable costs. This study can therefore act as a benchmark for other similar PICU’s and for planning cost minimisation trials.

We acknowledge a few limitations of our study. First, the time bound nature of the study dictated a small sample size and limited period of evaluation. Second, health system cost was apportioned uniformly for each patient and was not calculated individually as per their severity of illness and different procedures required (intracranial pressure monitoring, haemodialysis or continuous renal replacement therapy). Third, cost of liaison consultation has not been included in the costing process of the study. Lastly, since cost assessment was done from a single PICU, a sensitivity analysis could not be done with respect to delivery strategy.

## Conclusions

Pediatric ICU health system costs are 3.8 times more than the Out of Pocket expenditure in a public sector hospital. Severe illness, need for ventilation and duration of mechanical ventilation and longer ICU stay, were associated with increased costs of PICU care. This study can guide setting of the reimbursement package rates under AB-PMJAY. Tertiary level intensive care in India is far less expensive than developed countries. Intensive care in public sector is affordable compared to corporate sector hospitals.

## Abbreviations

PICU: Pediatric intensive care unit
OOP: Out of pocket expenditure
IQR: Inter quartile range
PRISM: Pediatric risk of mortality sore
AB-PMJAY: Ayushman Bharat – Pradhan Mantri Jan Arogya Yojana
ECMO: Extra corporeal membrane oxygenation
SPSS: Statistical Package for Social Sciences
PSI: Physiological Stability Index
HTAIn: Health Technology Assessment India
LMIC: Low middle-income countries
HBP: Health benefit package
CHSI: Cost of Health Services in India
